# Comparison of Gaze Behavior During a Visual Search Task in a Virtual Reality Environment Between Individuals With Alzheimer’s Disease and Healthy Older Adults

**DOI:** 10.7759/cureus.113761

**Published:** 2026-07-31

**Authors:** Tadatoshi Inoue, Takashi Nakamura, Lisa Senba, Jin Narumoto

**Affiliations:** 1 Department of Rehabilitation, Faculty of Health Sciences, Division of Occupational Therapy, Wakayama Professional University of Rehabilitation, Wakayama, JPN; 2 Faculty of Education, University of Teacher Education Fukuoka, Fukuoka, JPN; 3 Faculty of Health Sciences, Kumamoto Health Science University, Kumamoto, JPN; 4 Department of Psychiatry, Kyoto Prefectural University of Medicine, Kyoto, JPN

**Keywords:** alzheimer's disease, eye movement, shopping, virtual reality, visual search

## Abstract

Introduction: Decline in spatial attention is a core feature of Alzheimer's disease (AD) that directly impairs everyday visual search behavior, such as locating items during shopping. Japan's rapidly aging population has led to a growing prevalence of dementia, with AD being the most common subtype. The use of virtual reality (VR) enables standardized cognitive assessment in ecologically valid settings.

Methods: A total of 25 individuals with AD and 30 healthy controls (HC) aged 65 years or older performed a visual search task presented via a head-mounted display (HMD; VIVE Pro Eye, HTC Corporation, Taoyuan, Taiwan) showing a VR simulation of a supermarket beverage section captured with a 360° camera. Participants were instructed to locate a lemon tea product within 30 seconds. Eye movements were recorded binocularly at 120 Hz. The viewing field was divided into a 48×48 grid; fixation duration and proportion in the central region (columns 20-27, rows 24-33) were extracted and compared between groups using independent samples t-tests. The chi-square test of independence was used to compare the rate of successful target identification.

Results: Compared with HC, the AD group showed significantly longer central fixation duration (11.63 s vs. 6.66 s, p = .009) and a higher central fixation proportion (38.55% vs. 22.06%, p = .009). None of the AD participants identified the target within the time limit, compared with 15 of 30 (50%) HC participants (χ²(1) = 17.19, p < .001, φ = −0.559).

Conclusion: Individuals with AD demonstrated a restricted gaze pattern characterized by central fixation bias during VR-based visual search, reflecting impaired exploratory eye movements and narrowed visual search range. These findings suggest that VR-based gaze analysis may serve as a useful tool for evaluating functional visual cognition in AD and that consideration of these gaze characteristics may inform the design of more accessible retail environments and support daily functioning and social participation.

## Introduction

Japan is experiencing a continuous rise in the number of dementia patients in parallel with its aging population. A 2022 survey estimated the prevalence of dementia among adults aged 65 and older at 12.3%, with projections indicating that this figure will reach 5.84 million by 2040 [1]. Alzheimer's disease (AD) is the most common form of dementia, and in addition to memory and orientation impairments, deficits in visual cognition and attentional function are known to have a substantial impact on daily life [2]. In particular, declines in visual cognition and spatial attention have received increasing attention as key factors that directly impair everyday search behavior, such as locating a desired object [3]. Instrumental Activities of Daily Living (IADL), including shopping and financial management, have been reported to become impaired at an early stage in AD [4]. In grocery shopping scenarios, the visual search behavior involved in locating a target product on a shelf requires a combination of complex cognitive functions, encompassing visual information processing, spatial attention allocation, and search strategy planning. In individuals with AD, impairment of these functions is presumed to make product identification increasingly difficult, thereby limiting opportunities for outings and social participation [5].

Eye movement measurement is a valuable method for objectively evaluating visual search behavior, enabling quantitative characterization of visual information processing through analysis of gaze point distribution and dwell time [6]. Prior research has also reported delayed saccades and abnormal fixation behavior in individuals with AD, highlighting associations between cognitive function and eye movements [7]. Nevertheless, studies that have examined gaze behavior in individuals with AD in the context of a visual search task simulating an actual purchasing scenario remain limited [8]. In recent years, advances in virtual reality (VR) technology have made it possible to reproduce near-realistic environments under safe, standardized conditions, facilitating cognitive assessments that closely reflect everyday life. VR-based assessment permits behavioral observation even in situations where real-environment measurement is difficult, and its application to dementia assessment holds considerable promise [9,10].

Against this background, the present study employed VR technology to create a visual search task reproducing a supermarket beverage aisle, with the aim of comparing gaze behavior, specifically central fixation duration, central fixation proportion, and target-detection success, between individuals with AD and healthy older adults. We hypothesized that, compared with healthy older adults, individuals with AD would show a more restricted, centrally biased gaze pattern and a lower rate of successful target detection. This exploratory, cross-sectional comparative study is intended to characterize gaze behavior differences associated with AD; it is not designed as a validation study of a diagnostic or screening tool. The findings may, in the future, help inform the development of cognitive assessment methods and the design of more accessible store environments for people with AD, although such applications lie beyond the scope of the present study design.

## Materials and methods

Participants

The study included 25 individuals with AD and 30 healthy older adults who provided informed consent. Exclusion criteria were individuals unable to view images displayed on the head-mounted display (HMD) due to visual acuity or visual field impairments and those who did not provide consent. AD was operationally defined as a clinical diagnosis confirmed by each participant's own treating physician in the course of routine care; because participants were recruited from real-world clinical and day-service settings, the specific formal diagnostic criteria applied by individual physicians were not systematically recorded. Disease severity was not classified using a standardized instrument such as the Clinical Dementia Rating (CDR); the Mini-Mental State Examination (MMSE; administered to AD participants only) was used as a general index of global cognitive status rather than a formal severity classification.

Healthy controls did not undergo a structured cognitive screening instrument; cognitive normalcy was inferred from independent participation in community-based nursing-care prevention classes and the absence of a dementia diagnosis. Beyond the exclusion criterion of visual acuity/visual field impairment precluding HMD use, visual/neurological comorbidities and medication use were not systematically collected via a structured checklist; general health and lifestyle status were captured using the Latter-Stage Elderly Questionnaire (LSEQ). No participants in either group had a documented history of MCI. No a priori sample-size calculation was performed; the sample (n = 25 AD, n = 30 HC) comprised all participants for whom complete data were obtained during the recruitment period. All enrolled participants completed the five-point calibration procedure successfully, and none were excluded due to calibration failure. Participants were recruited from attendees of nursing-care prevention classes held in their local neighborhoods and users of adult day-service facilities. The eligibility criterion for age was 65 years or older. Recruitment for the nursing-care prevention classes was conducted with the cooperation of administrative bodies and local community leaders. The day-service facilities were those with whom a prior collaborative relationship had been established.

Ethical considerations

This study was conducted with approval from the Research Ethics Committee of Heisei College of Health Sciences (approval number: R07-06). The purpose and content of the study were explained to all participants, and written and verbal explanations were provided regarding the following: the right to decline participation, the right to withdraw consent after enrollment, guarantees of safety, anticipated benefits and risks, privacy management, protection of personal information, and the absence of any financial burden or compensation. Participants were enrolled only after written consent was obtained from both the individuals themselves and their legally authorized representatives. This study was supported by a Grant-in-Aid for Scientific Research from the Japan Society for the Promotion of Science (JSPS KAKENHI, Grant Number 24K05354). No funding or materials were received from any collaborative institutions. Conflicts of interest at each institution have been managed through conflict of interest management procedures, and no factors that could influence the results or interpretation of this research were identified.

Measurements

Basic demographic information, including age and sex, was collected from all participants. Lifestyle was assessed using the LSEQ, a 15-item instrument that enables comprehensive evaluation of health status, taking into account characteristics specific to older adults, such as frailty, and has demonstrated reliability and validity [11]. As the individual items of this questionnaire carry no assigned point values, responses indicating unfavorable conditions were converted to an ordinal scale for analysis purposes, with points assigned as shown in Table 1. Eye movement assessment involved recording fixation points along with their duration and proportion. Cognitive function was assessed using the MMSE-Japanese (MMSE-J). MMSE was administered only to participants with AD.

**Table 1:** Questionnaire for late-stage elderly individuals This questionnaire consists of 15 items in total. For each item, a response indicating a higher risk of frailty is scored as 1 point, while all other responses are scored as 0 points, and a total score is calculated. Based on previous studies, a total score of 4 or more was considered to indicate a risk of frailty.

No	Question	Response	
		0 points	1 point
1	How would you rate your current health status?	Good (Fairly good/Average)	Not very good (Poor)
2	Are you satisfied with your daily life?	Satisfied (Fairly satisfied)	Somewhat dissatisfied (Dissatisfied)
3	Do you eat three proper meals a day?	Yes	No
4	Have hard foods become more difficult to eat compared to six months ago? (Examples: dried squid, pickled daikon)	No	Yes
5	Do you sometimes choke on tea, juice, or other beverages?	No	Yes
6	Have you lost 2-3 kg or more in body weight over the past 6 months?	No	Yes
7	Do you feel that your walking speed has slowed compared to before?	No	Yes
8	Have you fallen during the past year?	No	Yes
9	Do you exercise (e.g., walking) at least once a week?	Yes	No
10	Have people around you told you that you often repeat yourself or are forgetful?	No	Yes
11	Do you sometimes not know what today's date is?	No	Yes
12	Do you smoke?	No (never or quit)	Yes (currently smoking)
13	Do you go out at least once a week?	Yes	No
14	Do you regularly spend time with family or friends?	Yes	No
15	Do you have someone nearby you can consult when you feel unwell?	Yes	No

Eye movement measurement

The beverage aisle of a supermarket was filmed using an Insta360 camera (Arashi Vision Inc., Shenzhen, China). The recorded footage was presented via a VR HMD, the VIVE Pro Eye (HTC Corporation, Taoyuan, Taiwan), and eye movements were recorded during viewing.

Eye movements were recorded binocularly at a sampling rate of 120 Hz using the built-in pupil-corneal reflection method via infrared cameras. Prior to the experiment, a five-point calibration procedure was performed for each participant. Fixation was defined as the gaze remaining within a designated area for a minimum of 300 milliseconds. Gaze-position preprocessing and the handling of missing or invalid gaze samples (e.g., due to blinks or tracking loss) were performed by the proprietary software embedded in the eye-tracking system (VIVE Pro Eye, in combination with analysis software provided by Business Engineering Corporation). The specific preprocessing algorithm and missing-data-handling criteria are proprietary to the vendor and are not disclosed for intellectual property reasons.

**Figure 1:**
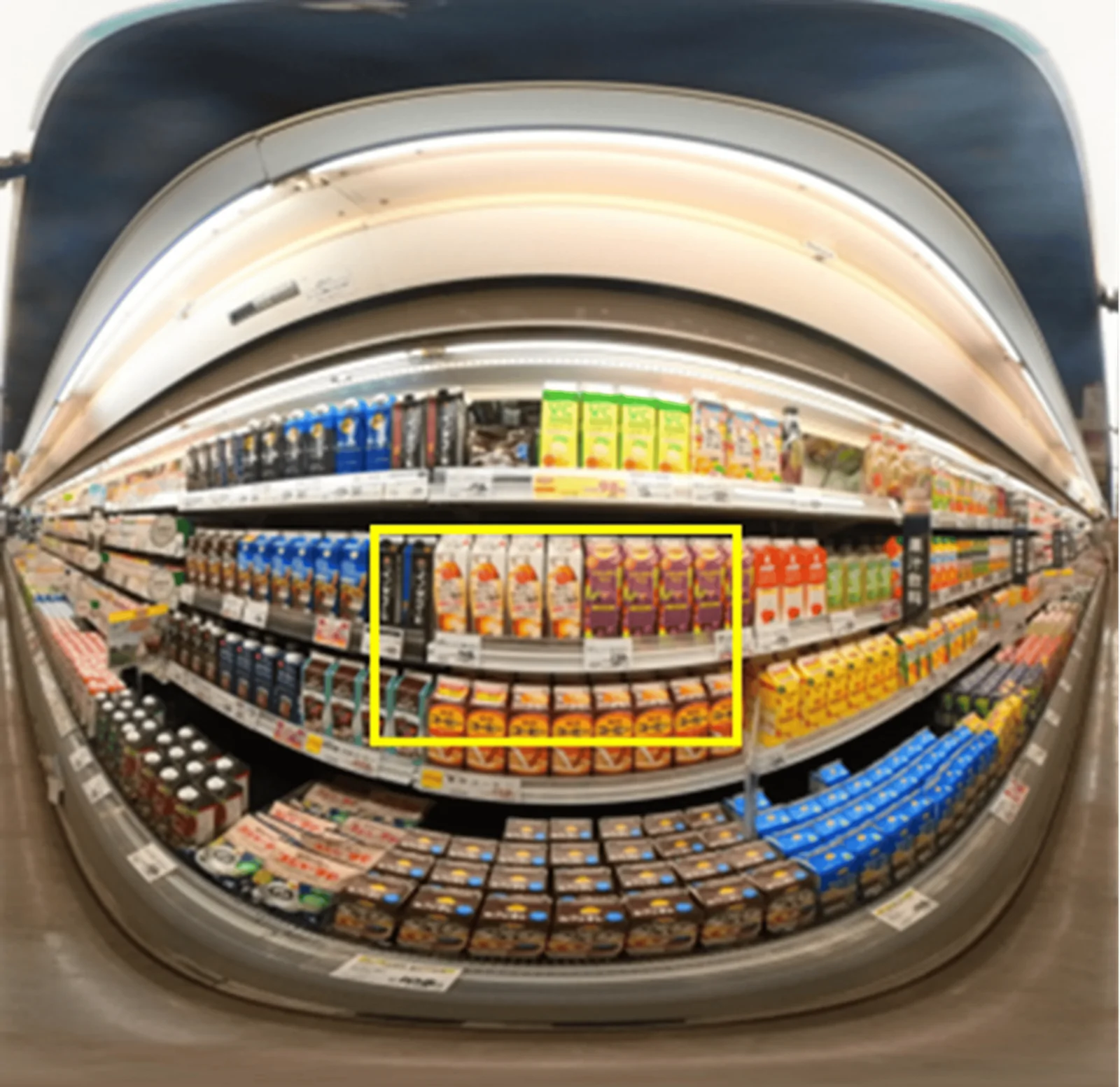
Representative scene of the HMD-based visual stimulus The image displays a flat projection of a single viewpoint taken from a 360-degree camera recording. The yellow box delineates the target area (the area of interest) within the refrigerated shelf. This scene was presented via a head-mounted display (HMD) to provide an immersive shopping environment for the subjects.

For analysis, the recorded footage was divided into a grid of 48 rows (1-48) × 48 columns (1-48), yielding 2,304 regions in total, and the dwell time of fixation points within each region was calculated. To compare fixation behavior between individuals with AD and healthy older adults, dwell times within the region corresponding to the central portion of the image, specifically columns 20-27 (horizontal) and rows 24-33 (vertical), were extracted (Figure 1). This region was operationally defined as the geometric center of the 48×48 grid, set a priori and independent of the observed gaze data. No established, externally validated criterion exists for delineating a "central" region of interest in VR-based visual search paradigms; prior work on the useful field of view indicates that its spatial extent varies considerably with attentional load and task demands, and, to our knowledge, no directly comparable VR-based visual search study in AD has reported a standardized definition of a central gaze region. This operational, literature-independent definition is discussed as a limitation below. Before the footage presentation, participants were instructed, "Please look for lemon tea" and "Please let us know when you have found it." The footage was presented for 30 seconds, with a single trial administered. Discovery of the lemon tea was defined as the participant verbally reporting that they had found it while their gaze fixation point was located on the position of the lemon tea.

Statistical analysis

Between-group differences for each measurement were analyzed using independent samples t-tests. The association between cognitive function group and whether the target object was discovered was examined using a chi-square test of independence. For the chi-square test, adjusted residuals of ≥2 or ≤−2 were used as the criterion for determining whether observed cell frequencies were high or low. Data are presented as mean (standard deviation) for t-tests and as the number of participants (adjusted residuals) for the chi-square test of independence. All analyses were performed using IBM SPSS Statistics for Windows, Version 31 (Released 2025; IBM Corp., Armonk, New York), with a significance level set at less than 5%.

## Results

Participant characteristics are presented in Table 2, and between-group comparisons for each measurement are shown in Table 3. This study examined the characteristics of individuals with AD by measuring age, care level, LSEQ scores, and visual search behavior in AD patients attending day-service facilities and community-dwelling healthy older adults.

**Table 2:** Characteristics of participants (n=55) Values are presented as mean ± standard deviation (minimum–maximum). MMSE: Mini-Mental State Examination. Care level: level of long-term care required. LSEQ: Latter-Stage Elderly Questionnaire. Central image fixation duration: total fixation duration on the central area of the image (sec). Central image fixation ratio: proportion of fixation points directed to the central area of the image out of all fixation points (%).

Item	Sample	Minimum	Maximum	Mean	SD
Age (years)	Total (n=55)	65	93	82.05	7.08
MMSE-J	AD group (n=25)	6	26	17.76	4.47
Care level	AD group (n=25)	1	3	1.4	0.58
LSEQ	Total (n=55)	0	10	4.13	2.4
Central image fixation duration (sec)	Total (n=55)	0	29	8.92	9.84
Central image fixation ratio (%)	Total (n=55)	0	96.6	29.55	22.69

**Table 3:** Comparison of each measurement variable between the Alzheimer's disease (AD) group and the healthy older adult group (n=55) Independent samples t-test: *p < 0.05, **p < 0.01. Values are presented as mean ± standard deviation. An independent samples t-test was used for comparisons between the two groups. AD: Alzheimer's disease. LSEQ: Latter-Stage Elderly Questionnaire. Central image fixation duration: total fixation duration on the central area of the image (sec). Central image fixation ratio: proportion of fixation points directed to the central area of the image out of all fixation points (%). Effect size (d): effect size calculated using Cohen's d. **p < 0.01.

Item	AD group (n=25)		Healthy older adults (n=30)		t	df	p	Effect size (d)
	Mean	SD	Mean	SD				
Age (years)	84.76	6.13	79.8	7.12	2.78	52.90	.008**	0.74
LSEQ	5.8	2.02	2.73	1.7	6.02	47.10	< .001**	1.66
Central image fixation duration (sec)	11.63	7.8	6.66	5.02	2.75	39.50	.009*	0.77
Central image fixation ratio (%)	38.55	25.87	22.06	16.64	2.75	39.50	.009*	0.77

The mean age was 84.76 years in the AD group and 79.80 years in the healthy older adult group, with the AD group being significantly older (t = 2.78, df = 52.9, p = .008, d = 0.74). LSEQ scores were 5.80 in the AD group and 2.73 in the healthy older adult group, with the AD group showing significantly higher scores (t = 6.02, df = 47.1, p < .001, d = 1.66). Central image fixation duration was 11.63 seconds in the AD group and 6.66 seconds in the healthy older adult group, with the AD group demonstrating significantly longer fixation times (t = 2.75, df = 39.5, p = .009, d = 0.77).

The proportion of fixation points in the central image region was 38.55% in the AD group and 22.06% in the healthy older adult group, with the AD group showing a significantly higher proportion (t = 2.75, df = 39.5, p = .009, d = 0.77). Because central fixation proportion was calculated as central fixation duration divided by the total trial duration (a near-constant approximately 30-second window across participants), the two measures are near-perfectly collinear; consequently, their independent samples t-test statistics are expected to be highly similar. Both analyses were independently re-verified against the raw per-participant fixation data.

These findings indicate that compared to healthy older adults, the AD group was older, showed greater health decline, and exhibited fixation behavior that was biased toward the central region of the visual display during a simulated shopping task in a VR environment.

Regarding the number of participants who successfully identified the target product within the allotted time, 0 participants in the AD group and 15 participants in the healthy older adult group located the item (Table 4). The chi-square test of independence revealed a significant association between group and discovery rate (χ²(1) = 17.19, p < .001, φ = −0.559). Adjusted residual analysis further indicated that the healthy older adult group had a significantly higher rate of target discovery (adjusted residual = 4.1), while the AD group showed a significantly higher rate of non-discovery (adjusted residual = 4.1).

**Table 4:** Comparison of target object detection between the Alzheimer's disease (AD) group and the healthy older adult group (n=55) Frequency (adjusted residual): **p < 0.01; χ² test for independence. Values are presented as frequency (adjusted residual). A chi-square test for independence was used for comparisons between groups. AD: Alzheimer's disease. Effect size (φ): effect size calculated using the phi coefficient. **p < 0.01.

	AD group (n=25)	Healthy older adults (n=30)	p-value	Effect size (φ)
Detected	0 (−4.1)	15 (4.1)	< .001**	−0.559
Not detected	25 (4.1)	15 (−4.1)		

## Discussion

In the present study, a visual search task requiring participants to locate lemon tea in a VR environment revealed that the AD group exhibited a characteristic pattern of fixation concentrated toward the center of the screen compared to healthy older adults. This finding was associated with impairments in both "visual information processing" and "search strategy" in individuals with AD; however, because age and other confounders were not statistically controlled, a causal relationship between AD pathology and this gaze pattern cannot be established from the present design.

Polden et al. [12] investigated saccadic initiation latency in response to gaze targets presented on a screen in 32 individuals with AD, 47 with mild cognitive impairment (MCI), 96 healthy older adults, and 44 healthy young adults, reporting that those with reduced cognitive function demonstrated delayed initiation of gaze shifts. Similarly, Eraslan et al. [13] measured eye movements and comprehensive cognitive function in 30 individuals with AD, 34 with amnestic MCI (aMCI), and 32 healthy controls and examined the relationship between cognitive function and saccadic initiation latency. Their results indicated that individuals with lower cognitive function showed delayed saccadic initiation. Collectively, these findings suggest that the sustained engagement and disengagement of attention toward a fixation target represent critical cognitive operations involving inhibitory control and that individuals with AD require more time to disengage attention. The pronounced central fixation bias observed in the AD group suggests a narrowing of the visual search range. While healthy older adults efficiently explored the entire screen by utilizing peripheral vision, individuals with AD appeared unable to make large eye movements to sample information across the display, resulting in an inefficient search pattern that precluded discovery of the lemon tea.

Visual attention has been reported to be regulated by the posterior parietal cortex and frontal cortical regions [14-16]. The visual system comprises a ventral visual pathway connecting the occipital and temporal lobes, responsible for processing object color and shape, and a dorsal visual pathway connecting the occipital and parietal lobes, responsible for determining object location and motion [17]. Visual search is accomplished through a cooperative network that additionally involves top-down control by the frontal lobe. It is therefore considered that in individuals with AD, dysfunction of the cortex, including the parietal and frontal lobes, impairs attentional allocation and spatial processing, resulting in a narrowed visual search range, biased exploration patterns, an inability to fully utilize visual information, and difficulty in achieving a holistic grasp of the visual scene.

Limitations

This study examined the characteristics of fixation points during a visual search task according to cognitive status. The AD group was significantly older and had a significantly higher (worse) LSEQ score than the healthy older adult group, and neither variable was statistically controlled in the present analysis. It is therefore possible that age, general health/frailty status, or other unmeasured factors, rather than AD pathology per se, contributed in part to the observed differences in fixation behavior and target-detection rate.

The relatively small and unbalanced sample sizes (n = 25 AD, n = 30 HC) further limit the statistical power and generalizability of these findings. In addition, the AD and HC groups were recruited from different settings (day-service facility attendees and community-based nursing-care prevention class attendees, respectively), which introduces a risk of selection bias that cannot be ruled out. Future studies should recruit age- and health-status-matched samples and apply covariate-adjusted statistical models (e.g., ANCOVA) to clarify the independent contribution of AD to gaze behavior.

The central region of interest used in this study was operationally defined as the geometric center of the visual field rather than derived from an established, externally validated criterion, as no standardized definition of a "central" gaze region could be identified in the prior literature; this somewhat arbitrary operationalization is therefore an additional limitation. Future studies should incorporate a whole-screen, heat-map-based gaze-distribution analysis to corroborate the central-fixation-bias finding independent of this specific ROI. In addition, because the central fixation proportion is mathematically derived from the central fixation duration divided by a near-constant trial duration, these two measures are highly collinear and should not be interpreted as independent lines of evidence; they are reported together for completeness and interpretability (duration in absolute time, proportion in relative terms) rather than as separate corroborating findings.

Furthermore, as attentional function was not formally assessed, the presence and magnitude of between-group differences in attention remain unclear. Finally, gaze preprocessing and missing-data handling were performed by proprietary, vendor-supplied software whose internal algorithm could not be disclosed, which may limit exact technical reproducibility by laboratories using different eye-tracking systems. Future research should incorporate attentional assessments and integrate those findings with the present results.

Finally, because this study was conducted under the specialized conditions of a VR environment, the experimental setup involving a display-based VR presentation may have influenced the results. Individuals with dementia may experience greater difficulty with depth perception and spatial understanding in virtual environments such as VR compared to real-world settings. It is also possible to interpret the central fixation bias as a manifestation of anxiety or a freezing response caused by excessive concentration in response to this unfamiliar spatial environment. Future studies should establish conditions more closely approximating an actual retail environment, including improvements to display resolution and the incorporation of ambient sound.

## Conclusions

This exploratory study compared fixation behavior between individuals with AD and healthy older adults using a visual search task administered in a VR environment. The AD group exhibited fixation points concentrated toward the center of the screen, a pattern that was associated with lower target-detection success. However, because age, general health status, and recruitment setting differed between groups and were not statistically controlled, these findings should be interpreted as a preliminary, hypothesis-generating association rather than evidence of a direct effect of AD pathology. If replicated in age- and health-matched samples using covariate-adjusted analyses, this central fixation bias could have implications for understanding difficulties locating objects in daily life and, potentially, for informing the design of more accessible product displays and social environments for people with AD.

## References

[1] (2026). Ministry of Health, Labour and Welfare: Future projections of the number of older adults with dementia and mild cognitive impairment (MCI) and their prevalence rates (Article in Japanese). https://www.mhlw.go.jp/content/001279920.pdf.

[2] Safiri S, Ghaffari Jolfayi A, Fazlollahi A (2024). Alzheimer's disease: a comprehensive review of epidemiology, risk factors, symptoms diagnosis, management, caregiving, advanced treatments and associated challenges. Front Med (Lausanne).

[3] Pereira ML, Camargo MV, Bellan AF (2020). Visual search efficiency in mild cognitive impairment and Alzheimer's disease: an eye movement study. J Alzheimers Dis.

[4] Edemekong PF, Bomgaars DL, Sukumaran S, Schoo C (2022). Activities of daily living. StatPearls [Internet].

[5] Ramzaoui H, Faure S, Spotorno S (2018). Alzheimer's disease, visual search, and instrumental activities of daily living: a review and a new perspective on attention and eye movements. J Alzheimers Dis.

[6] Readman MR, Polden M, Gibbs MC, Wareing L, Crawford TJ (2021). The potential of naturalistic eye movement tasks in the diagnosis of Alzheimer's disease: a review. Brain Sci.

[7] Eraslan Boz H, Koçoğlu K, Akkoyun M, Tüfekci IY, Ekin M, Akdal G (2024). Visual search in Alzheimer's disease and amnestic mild cognitive impairment: an eye-tracking study. Alzheimers Dement.

[8] Akkoyun M, Koçoğlu K, Eraslan Boz H, Tüfekci IY, Ekin M, Akdal G (2024). Visual search for real-world scenes in patients with Alzheimer's disease and amnestic mild cognitive impairment. Brain Behav.

[9] Tarnanas I, Schlee W, Tsolaki M, Müri R, Mosimann U, Nef T (2013). Ecological validity of virtual reality daily living activities screening for early dementia: longitudinal study. JMIR Serious Games.

[10] Gausemel Å, Filkuková P (2025). Innovations in dementia screening: a systematic review and meta-analysis of virtual reality assessments. Front Psychol.

[11] Japan Geriatrics Society (2026). Japan Geriatrics Society. Manual for the Latter-Stage Elderly Questionnaire. https://www.jpn-geriat-soc.or.jp/tool/manual.html.

[12] Polden M, Wilcockson TD, Crawford TJ (2020). The disengagement of visual attention: an eye-tracking study of cognitive impairment, ethnicity and age. Brain Sci.

[13] Eraslan Boz H, Koçoğlu K, Akkoyun M (2022). The influence of stimulus eccentricity on prosaccade outcomes in patients with Alzheimer's disease dementia at an early stage and amnestic mild cognitive impairment. J Clin Exp Neuropsychol.

[14] Thut G, Nietzel A, Pascual-Leone A (2005). Dorsal posterior parietal rTMS affects voluntary orienting of visuospatial attention. Cereb Cortex.

[15] Bisley JW, Goldberg ME (2003). Neuronal activity in the lateral intraparietal area and spatial attention. Science.

[16] Wang M, Yu B, Luo C (2020). Evaluating the causal contribution of fronto-parietal cortices to the control of the bottom-up and top-down visual attention using fMRI-guided TMS. Cortex.

[17] Nagasaki S, Suzuki T (2022). Gold Master Textbook: Higher Brain Dysfunction Occupational Therapy, 3rd Edition (Article in Japanese). https://www.medicalview.co.jp/catalog/ISBN978-4-7583-2045-0.html.

